# Public health and valorization of genome-based technologies: a new model

**DOI:** 10.1186/1479-5876-9-207

**Published:** 2011-12-05

**Authors:** Jonathan A Lal, Tobias Schulte in den Bäumen, Servaas A Morré, Angela Brand

**Affiliations:** 1 Institute for Public Health Genomics, Department of Genetics & Cell Biology, CAPHRI, Faculty of Health, Medicine and Life Sciences, Maastricht University, Universiteitssingel 5, 6229 ES Maastricht, The Netherlands; 2 Laboratory of Immunogenetics, Department of Pathology, VU University Medical Centre, De Boelelaan 1117, 1081 HV Amsterdam, The Netherlands; 3 Institute for Public Health Genomics, Department of Genetics & Cell Biology, Research Institutes CAPHRI and GROW, Faculty of Health, Medicine and Life Sciences, Maastricht University, Universiteitssingel 5, 6229 ES Maastricht, The Netherlands

**Keywords:** Technology Transfer, Health Technology Assessment, Public Health Genomics, Health Needs Assessment, Health Impact Assessment, Valorization, Translational Research, Healthcare, Health Policy, Genomics

## Abstract

**Background:**

The success rate of timely translation of genome-based technologies to commercially feasible products/services with applicability in health care systems is significantly low. We identified both industry and scientists neglect health policy aspects when commercializing their technology, more specifically, Public Health Assessment Tools (PHAT) and early on involvement of decision makers through which market authorization and reimbursements are dependent. While Technology Transfer (TT) aims to facilitate translation of ideas into products, Health Technology Assessment, one component of PHAT, for example, facilitates translation of products/processes into healthcare services and eventually comes up with recommendations for decision makers. We aim to propose a new model of valorization to optimize integration of genome-based technologies into the healthcare system.

**Methods:**

The method used to develop our model is an adapted version of the Fish Trap Model and the Basic Design Cycle.

**Results:**

We found although different, similarities exist between TT and PHAT. Realizing the potential of being mutually beneficial justified our proposal of their relative parallel initiation. We observed that the Public Health Genomics Wheel should be included in this relative parallel activity to ensure all societal/policy aspects are dealt with preemptively by both stakeholders. On further analysis, we found out this whole process is dependent on the Value of Information. As a result, we present our LAL (Learning Adapting Leveling) model which proposes, based on market demand; TT and PHAT by consultation/bi-lateral communication should advocate for relevant technologies. This can be achieved by public-private partnerships (PPPs). These widely defined PPPs create the innovation network which is a developing, consultative/collaborative-networking platform between TT and PHAT. This network has iterations and requires learning, assimilating and using knowledge developed and is called absorption capacity. We hypothesize that the higher absorption capacity, higher success possibility. Our model however does not address the phasing out of technology although we believe the same model can be used to simultaneously phase out a technology.

**Conclusions:**

This model proposes to facilitate optimization/decrease the timeframe of integration in healthcare. It also helps industry and researchers to come to a strategic decision at an early stage, about technology being developed thus, saving on resources, hence minimizing failures.

## Background

Over time we have seen enormous transition of genome-based/life science research from the lab to products and technologies [1-3] on the market [3-6] as a result of knowledge valorization and spin-offs [2,3,7,8]. This can be attributed to the concept of translational research, which is the effective translation of new knowledge, mechanisms and techniques generated by advances in basic science research into new approaches for prevention, diagnosis and treatment of disease essential for improving health [9]. However, we notice that the timely translation of genome-based technologies to commercially feasible products with practical applicability or direct implementation in health care systems is quite low [10]. This is evident by the large amount of data present in literature [11], patents [12,13] and the market [3,5] compared to what actually is being used in hospitals [10,14]. We identify, based on our experience as well as derivatively, three phases of translation. The first phase includes translation from lab to industrial application (see T1 of Khoury *et al*.) [15], the second phase being from industry to market penetration [16] and the third phase being, shift from the market to integration in health policy (see T3 of Khoury *et al*.) [15]. We believe both academia and industry focus only on one or maximum two of the three translational phases.

**Figure 1:**
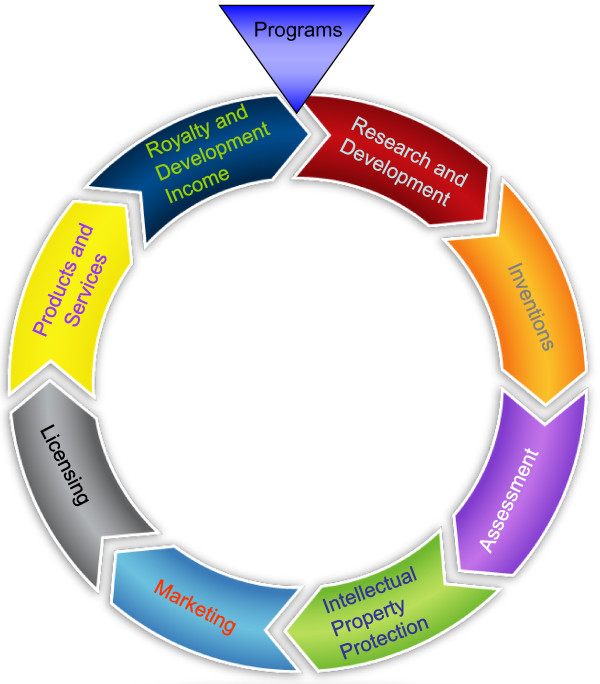
**The Technology Transfer Process**. The basics include the start from research and development based on market pull or push, successfully inventing a product based on that research, patenting the product, thereby marketing and licensing and going through the technology maturation process. Taken from the North Carolina Agricultural and Technical State University [18].

In addition, methodologies in place for translation generally focus on the first two phases or the last phase and as per our knowledge we have not seen a combination of the three, overlaps or jumps in general. For example, Technology Transfer (TT) mainly addresses the first two phases mentioned above. TT is seen as an activity of the migration of academic discoveries to useful application in the development of marketable products or processes [17]. TT can involve several steps from organization to organization and can have separate offices specialized in this activity. For example, universities have a TT office or valorization center responsible for TT activities of university research. Basically, the TT activity initiates from an invention of an innovative idea, through the development of the idea into a pilot to the creation of the technology based on the idea followed by patenting the technology in question and ends after the technology maturation process. The maturation process involves the return on investment and exit strategy of a company with respect to the technology concerned. A simplistic example can be seen from Figure 1 below [18]. TT is the most widely used activity [1] in business development of academic research and spin-offs [19]. It should be noted here however, TT can be considered as an activity, methodology, tool or technique depending on the user. However from the objective of our paper, we will consider this as an activity.

The most common procedures used by decision makers (DM) and health professionals in phase three mentioned above involve the traditional public health evaluation instruments [20], which include Health Needs Assessment (HNA), Health Technology Assessment (HTA) and Health Impact Assessment (HIA). HNA is a systematic method of reviewing the health issues facing a population, leading to agreed priorities and resource allocation that will improve health and reduce inequalities [20]. Correspondingly, HTA is a multidisciplinary process that summarizes information about the medical, social, economic and ethical issues related to the use of a health technology in a systematic, transparent, unbiased, robust manner. Its aim is to inform the formulation of safe, effective, health policies that are patient focused and seek to achieve best value [21]. This implies to interventions as well as HTA's involvement in appraisals. Thus, HTA is a powerful tool to inform policy making. According to the World Health Organization, HIA is a combination of procedures, methods and tools by which a policy, program, or project may be judged as to its potential effects on the health of a population, and the distribution of those effects within the population [22]. To summarize, HTA evaluates the performance of health care technologies, HIA assesses the effects of policies, programs or projects on the population's health and HNA identifies health priorities for a given population [20]. For our convenience, we will use the term Public Health Assessment Tools (PHAT) for the collective reference to HNA, HTA and HIA in the text.

It becomes apparent from above, that the translational phases 1-2 and 3 mentioned above are investigated by two separate entities, namely, the academia-industry infrastructure [23] and the governmental bodies respectively. This is the key reason why the two operate as separate functions. While TT aims to facilitate the translation of ideas into products, HTA for example, assesses the translation of products and services into healthcare. As per content, they both seem inseparable in the order stated, however to our knowledge have never been connected. As a result, there is a backlog of relevant technologies to be integrated in Public Health and healthcare in general in a timely as well as effective and efficient manner [10,24,25]. Even by the time a technology is introduced in the health care system, it is in a way deemed redundant as more effective and efficient technologies become available in the market. Redundancy is from the perspective of the potential of existing technologies on the market compared to the previously introduced one in the hospital. Efficiency measures whether healthcare resources are being used to get the best value for money [26]. Therefore, efficiency can be seen in terms of added value, speed and cost, although costs can vary. According to Ostrower [27], effectiveness has several varying definitions but one of the common ones used by institutions includes the component of 'having an impact'. On the other hand (clinical) effectiveness is the extent to which specific (clinical) interventions do what they are intended to do, i.e. maintain and improve the health of patients securing the greatest possible health gain from the available resources [28]. In other words, effectiveness and also in our case, refers to the achievement of the overall goal (the effect). We identify the non-synergy between TT and PHAT, as the bottleneck of technological integration in the health care.

This brings in the new field of Public Health Genomics which is the responsible and effective translation of genome-based knowledge and technologies into public policy and health services for the benefit of population health [29]. It is an emerging field with increased demands of integration of genome-based innovations i.e. genome-based knowledge and technologies, into Public Health due to the prior's increasing value and relevance in the latter. Within the concept of Public Health Genomics and keeping in mind the bottleneck mentioned above, we aim to propose a new model to facilitate valorization of genome-based technologies into the healthcare system in real time.

The term valorization can vary depending upon the user (say either the economist or entrepreneur). According to the Merriam-Webster online dictionary [30], one definition of valorize states 'to assign value or merit to'. On the other hand, the term valorization can be defined as the transformation of knowledge into concrete new products, services and processes [31]. Deriving from these two definitions and keeping in mind the definition of Public Health Genomics, valorization in Public Health Genomics can be seen as the 'process of realization' of relevant added value 'bioproducts' in the domain of Public Health for benefit of the population and healthcare systems. Here we consider realization in terms of understanding the importance, impact or potential benefit and implementing it. The term bioproduct may have varying definitions [32,33], here again based on the user or source. According to the working definition submitted to the British Columbia Bioproducts Working Group [34] with the background of agro-forestry, bioproducts are sustainable, environmentally friendly novel products, or products satisfying novel applications and generated from renewable (living) bio-resources based on technologically advanced eco-efficient conversion processes. Within our scope of usage focusing on integration of genome-based technologies/life sciences into healthcare, we define bioproducts as sustainable novel products or products satisfying novel health applications derived or inspired from genome-based information or technologies. We define a product as a system, device, technique or process or application. Genome-based technologies encompass all -omics initially deriving from the (Human) Genome.

## Methods

**Figure 2:**
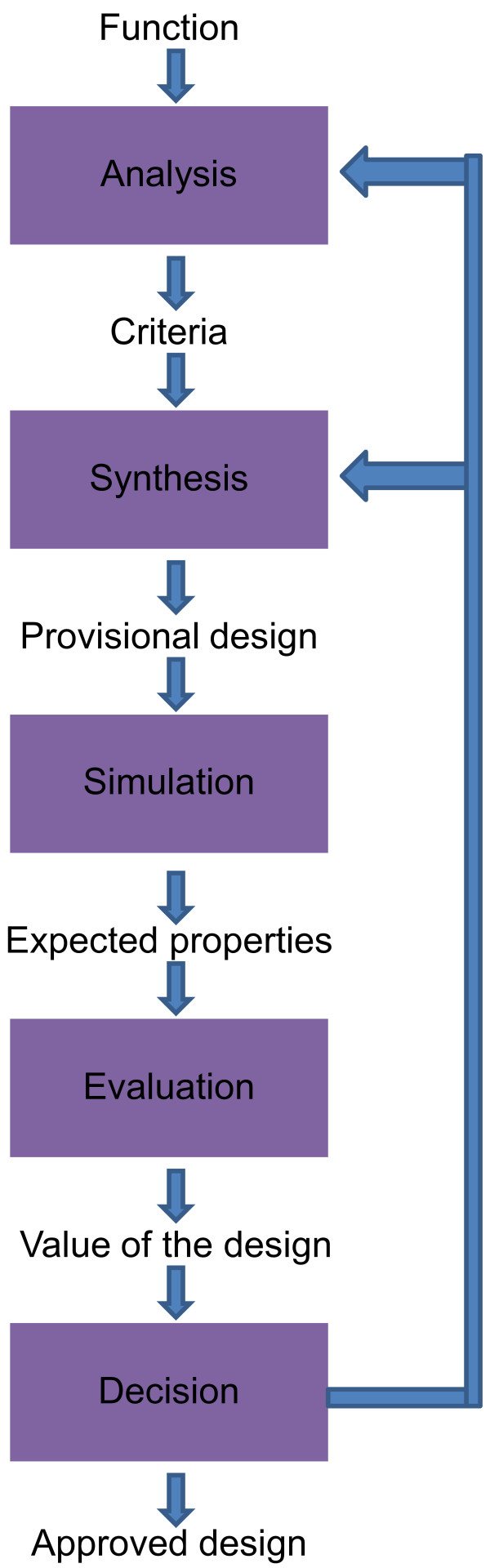
**The basic design cycle**. The most basic design methodology used. All design problems have one way or the other went through this design directly/indirectly. See text for description. Taken from Roozenburg *et al. *[35].

**Figure 3:**
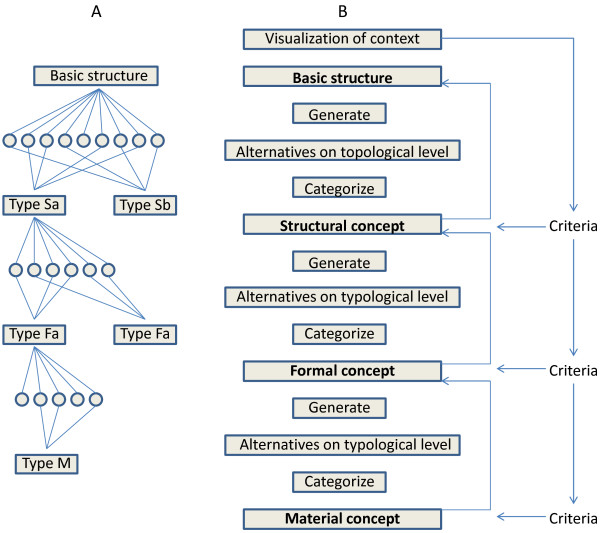
**The fish trap model**. (A) The convergence-divergence illustration. 'Sa and Sb' refer to structural concept a and structural concept b respectively. Similarly, 'Fa and Fb' refer to formal concept a and formal concept b respectively. 'M' refers to material concept. (B) the phase or step-wise illustration of the fish trap model. See text for description. Taken from the Delft Design guide [36].

**Figure 4:**
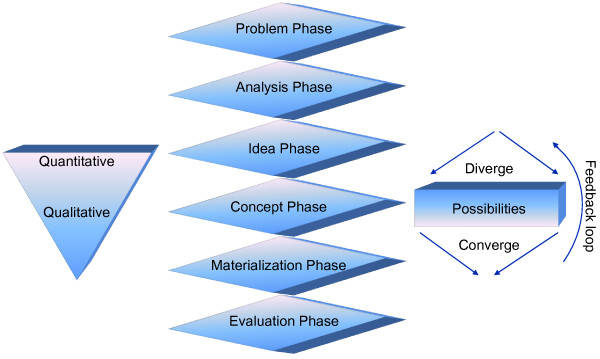
**The methodology: adapted from the Fish Trap Model and the Basic Design Cycle**. The first phase is called the 'Problem phase'. This phase deals with identifying the problem, which needs to be solved, and understanding it. The next phase is the 'Analysis phase'. In this phase we look at the problem in detail and analyze relevant data per se. Next comes the 'Idea phase'. This phase derives possible solutions based on the analysis phase coming up with the possible options, directions or ideas to proceed with and can overlap with the analysis phase. Following the idea phase is the 'Concept Phase'. From the list of options generated, based on merits, one or few are selected to work on further detail or conceptualize it. Thereafter the conceptualization, the product is materialized or brought forth and implemented. This is followed by the evaluation of the product. The whole process can have iterations and moves from a large set of options and narrows down to a single idea to be implemented. Adapted from the Basic Design Cycle [35] and the Fish Trap Model [36].

The methodology we used to develop the model is based on the Basic Design Cycle [35] and the Fish Trap model [36] as can be seen from Figure 2 and 3 respectively. The general methodologies mentioned above are used mainly in designing products for commercial exploitation. In our case, the methodologies have been adapted to fit our purpose, which can be seen in Figure 4 below. We use this adapted design technique to address a Public Health and health policy issue, the integration of genome-based technologies into healthcare in the concept of a new model. In the subsequent paragraphs we briefly explain the two models from which our methodology derived from followed by the derived methodology itself.

The Basic Design Cycle (BDC) is stated to be the most fundamental model of designing. The intended behavior or function of the product is the start of product design as can be seen from Figure 2. Broad statements on the function/properties (use) are made in order for the designer to know what needs to be designed. In the analysis phase the problems surrounding a new product idea are identified (also called the problem statement), thereby criteria are formulated from general to specific ones over iterations which the solution should satisfy. The next step is the development of a provisional design via the synthesis phase (combining things) [35]. This phase is based on human creativity and is considered the moment of externalization and description of an idea on any form. The next phase is simulation phase which is considered a deductive process. Simulation in essence is forming an image of the behavior and properties of the designed product through testing, reasoning, etc. and results in anticipation of the real properties or conditional predictions for the new product. The next phase is the evaluation in which the value of the design is established. This is based on comparison of previously mentioned set specific criteria (see Figure 2) and actual properties in the design. The last phase is the decision phase whether to continue or reconsider. There is the possibility that the designer may need to return to the analysis or synthesis with subsequent iterations and feedback loops [35].

The Fish Trap Model (FTM) (can be considered to be called as to catch a final solution) is a systematic process of designing a product form. As can be seen in the Figure 3, the fish trap is illustrated in two ways, (Figure 3A) to visualize the divergence and convergence as well as to show the occurrence of various solution types at each level [36]. Once possible variants are created through curiosity/possibilities, they are categorized according to solution type. Thereafter, one or more variants are chosen to be developed into a concept which indicates a specific solution type. Again, from here more concrete solutions are derived from the chosen variants thus diverging again. This narrows down to one or few options to go forth with [36].

As can be seen from Figure 3B, the FTM is represented in a step by step or phase format. Since the model pushes the designer to discover alternatives on 3 levels, namely topological, typological and morphological, it is considered a systematic process as can be seen in the figure. Following step by step Figure 3B, in order to develop the 'structural concept' the 'basic structure' functional components need to be defined in advance through 'visualization of context'. One can develop or 'generate' multiple variants with these components that may differ in their respective spatial placement. These divergent are then clustered (convergence) or 'categorize' and a representative variant are selected to be developed into a 'structural concept'. Again, selection of these structural concepts will be based on evaluation criteria and need to be put into context. For the development of a 'formal concept', one or more structural concepts are taken [36]. From these selected structural concepts, geometric constructions via sketching are made leading to different classes of form (generate step). These sketches are evaluated based on their viability and categorized into groups based on form, and improvements can be still made and evaluated. Thereafter, promising candidates are further developed into one or more 'formal concepts'. Similar to above, further materialization of one or more formal concepts is done with a diverging exploration process again looking at a detailed level at the solution [36].

Our methodology was adapted from the above two mentioned models. As can be seen in Figure 4 the first phase is the problem phase derived from the concept of the 'problem statement' of the analysis phase of the Basic Design Cycle (BDC). In our model the problem phase includes both stating the problem (statement) of the BDC as well as identification of the intended behavior or function of the product which in our case is a framework. This phase not only deals with identifying the problem which needs to be solved but as well as understanding it. As a result the intended function of the final solution is cleared from the beginning.

The next step in our methodology is the analysis phase which is derived partly from the remainder of the analysis phase in the BDC and the structural concept phase of the Fish Trap Model (FTM). From the latter, the basic functional components are taken into our analysis phase. In other words, we look at the problem and its potential components and factors in more detail and analyze the data. Additionally, from the BDC concept, our analysis phase also includes developing criteria for the analysis and can include possible iterations and feedback.

As a result of the analysis phase several options are generated/developed to go forward with, which we call the idea phase and is the next step in our methodology. The components of this phase are derived from the synthesis phase of the BDC which is based on human creativity (and describes the idea on any form) and the divergence concept (several possibilities) of the FTM. Also, from the FTM, the basic functional components previously defined in our analysis phase are developed into multiple variants and clustered forming a structural concept. This step is considered the externalization of several relevant ideas.

The concept phase is the follow-up step of the idea phase in our methodology and is based on the simulation step of the BDC to develop the provisional design and is a deductive process as one or more options are chosen from a list (in the idea phase) based on the context it can solve. Also, the concept phase influentially derives from the formal concept step of the FTM which include assessing one or more structural concepts from our idea phase as well as geometric sketches. From these sketches through iterations and feedback loops one final version is selected.

The above mentioned steps in our adapted methodology (see Figure 4) in line with the BDC and the FTM, initially quantify the data down to a single qualitative product or in our case, a model. This is achieved through iterations and feedback loop which include diverging and converging of data or components. In other words development initiates with several ideas and through the process and feasibility analysis narrows down from a quantity of data to one final option good in quality. The scope of our methodology includes and ends at the concept phase since based on the fact the model we propose is a theoretical framework. Nonetheless, validation or proof of principle and evaluation are logically the next steps although not within the scope of this paper. Therefore we keep these two points in the to-be final two steps of our methodology mentioned below.

Materialization phase will be the next step of our methodology although not utilized in the paper. This is derived directly from the materialization phase of the FTM and may include diverging exploration if necessary. The implementation of the product or in our case the model/framework is considered the completion of this phase. The final to-be phase is the evaluation phase which is a direct derivation of the decision phase of the BDC with a modified approach to evaluate rather than to come to a decision. However this evaluation can lead to further adaption of the model.

## Results

**Figure 5:**
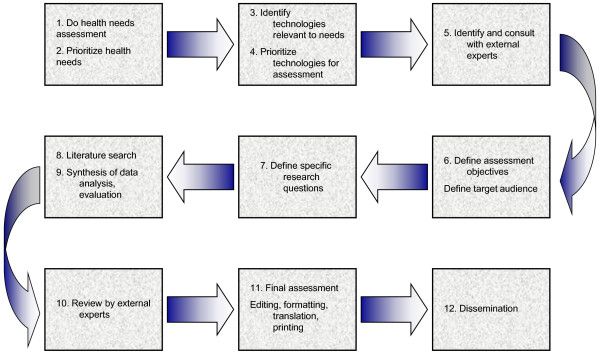
**A general outline of the HTA procedure**. The picture displays how HTA investigation initiates and the different steps involved followed by dissemination or recommendation to Government officials, for example whether or not to go forward with the technology in question in health policy implementation. Taken from the Canadian Coordinating Office for HTA [37].

On further analysis, we identified that the mainly used activity in transfer of academic knowledge to industry/market is TT [1,19]. On the other hand, the system used by decision makers to come to a decision regarding implementation of technologies in the healthcare system are the PHAT. For simplicity, we will take one example of the PHAT, i.e. HTA with references to other components when seemed appropriate. As known HTA is initiated by the need and identification of technologies which can reduce the burden of illness and through its steps, prioritize those with importance and relevance [37,38] and eventually come up with recommendations for DMs. Taking into account the HTA process [37] as can be seen in Figure 5 below, it has several steps. The TT activity however, similar to HTA in its complexity of dynamic steps, varies from organization to organization. For our case we will use the simple example, given by North Carolina Agricultural and Technical State University, [18] as shown in Figure 1 above. As we can see from the Figures 1 and 5, there are quite some similarities or overlaps between the two different approaches as well as per definitions of the two seem inseparable in the order stated.

However, after comparing Figure 1 and 5 above, it seems that TT should move in parallel with HTA rather than one after the other. Below are suggested relations between the two:

1. HNA/HTA initiates with the identification of current health needs and prioritizes accordingly. On the other hand, TT, kick-starts with research and development, which in part is motivated by the current needs in the market (market pull) or creates a need based on scientific data (market push). It should be noted here that literature suggests a significantly large amount of scientific data relevant and applicable to health [11] which is not implemented in the same [10], therefore justifies market push in health and medicine. The key word here being 'need' is the common ground of motivation for both TT and HNA/HTA initiation, suggesting the reason for both of them to start in parallel unlike what their respective definitions suggestion of one after the other. In other words, HNA and HTA identify the gaps in the needs of healthcare technology and its subsequent requirement of investigation respectively, and TT capitalizes on this need.

2. It should be stressed that consultation with external experts form a core component of HTA during the prioritizing of relevant technologies. Generally speaking, experts in the field develop the technology in the TT process, and with regard to medical technologies, take feedback from the end-consumers.

3. The core component of HTA is the assessment phase (including the economic, ethical, legal and social implications) of the technology in question and can also include Horizon Scanning (can be considered part of HNA and early stages of HTA) [39]. Horizon Scanning is defined as a systematic process for objectively evaluating the status of potential benefits of foreseeable technological developments based on contemporary research and evidence [40]. It also implies that it may be used as a type of SWOT (Strengths, Weaknesses, Opportunities and Threats) analysis [40]. Assessment of the feasibility of the invented technology is a given in TT before patenting or placing it in the market and this generally includes SWOT analysis. This also brings forth the HIA, where the impact of potential technology to be used is assessed, which also related to the TT's strategy before introducing the technology concerned in the market. It seems logical that TT should encompass Horizon Scanning within the aspect of HNA and HTA.

4. Another point is that both fields identify related stakeholders during the process.

It is not of the reasons above that the two (PHAT and TT) should start off together. Rather the essence of the story lies in the fact that both separate fields can and should benefit from each other and en-route streamline the integration process in order to properly address the whole innovation pipeline. Based on the authors' experience working with, in and around the Biotech industry, the bottleneck of technology integration is rooted in the fact that the industry neglects the policy aspects [41] (can include but not limited to market authorization, reimbursement and market penetrance) of technology which is an important factor in determining technology use in Public Health and healthcare at the end of the technology maturation process. Similarly, DMs only assess existing mature technologies to cover the aspects of current need, which results in the untimely introduction of relevant or upcoming technologies that can solve the 'need' issue more effectively and efficiently. With this flow of thought, the above mentioned points which suggest the anonymous inter-relations between TT and PHAT bring forth a new window of opportunity to use these pre-existing respective setups to expand the bottleneck for sustained flow of technological integration in the healthcare system through simultaneous initiation of these methods rather than one after the other.

Based on the above, our model should include relative parallel initiation of TT and PHAT with a sustainable infrastructure or network for the two to communicate bi-laterally and mutually benefit. On further research, we found that the Public Health Genomics Wheel (PHGW) [42], which describes Public Health components or areas that can be addressed by genome-based information and technologies, seems suitable to fit in our model. Furthermore, we identified that the Value of Information (VOI), the amount a person is willing to pay to come to a decision on a subject [43], seems appropriate for our model.

**Figure 6:**
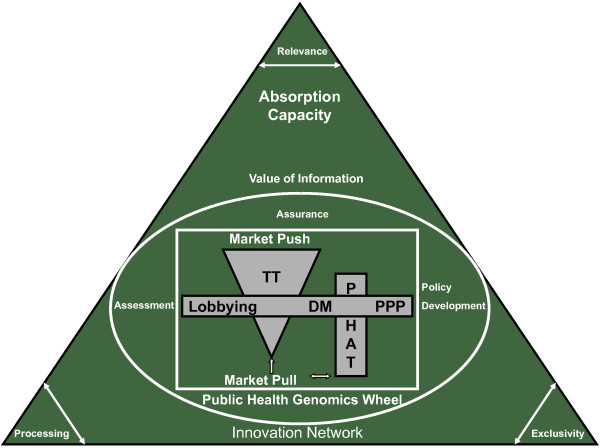
**The LAL Model: Learning Adapting Leveling**. The model's core consists of two components, the TT activity and the PHAT process (used by DMs). This model proposes bi-lateral communication, collaboration and advocating for relevant technologies during and between the (early-mid) TT activity and PHAT process both of which are driven by market pull/push (common denominator). This is achieved possibly through PPPs. The two components/entities (TT & PHAT) use the PHGW as a reference point to see that all policy and societal aspects are covered. This ongoing interaction through PPPs develops the innovation network for the current and future collaborations which is a network or platform through which common goals can be achieved. This innovation network is dependent on the VOI, i.e. the actual relevance (including added value) of the technology in question, the exclusivity (patent, license, etc.) of the technology to be integrated including possible hindering factors as a result, and the processing ability or understandability of user (say health professional or doctor, etc.) with respect to the technology and its impact. Over the process, the two core components (TT and PHAT), learn and adapt from each other within the innovation network and level out differences in approach and concept through PPPs (in essence learning-adapting-leveling) through feedback loops and iterations. This can be achieved over multiple or repetitive PPPs. Each network has a threshold of optimal capacity for such a learning curve, which we call the absorption capacity. The model indicates the right balance between profit and timely interventions.

As a result of our investigation, we present our model which we call the 'LAL Model' as can be seen in Figure 6 below and stands for 'Learning Adapting Leveling'. As we know, TT is driven by either market pull or market push and PHAT is driven by market pull. A technology (or market) push implies that a new invention is pushed through research and development, production and sales functions onto the market without proper consideration of whether or not it satisfies a user need [44]. In contrast, an innovation based upon market pull has been developed by the research and development function in response to an identified market need [44]. Also it is known that market push can lead to market pull and vice-versa. It should be noted here when talking in terms of PHAT market pull is not motivated due to commercialization although TT may recognize it in that way, but rather by requirement or health need. However, the (market) pull or need is the common ground which fuels both PHAT (in particular HNA/HTA) and TT respectively. When we talk about TT, it moves from quantitative to qualitative output. In other words, research and development initiates with several ideas and through the process and feasibility analysis, quantifies down to a single qualitative product to be commercialized. This is represented in our Figure 6 as TT being an inverse triangle. What also can be noticed is that the TT triangle and PHAT bar are standing in parallel, with the prior starting a bit earlier than the latter. TT identifies possibilities of commercialization via market pull/push based on sound scientific knowledge. The initial stages of TT start in the lab with research and development. At this stage, the research is motivated on market dynamics and cannot clearly identify with the PHAT's Health Needs Assessment; hence the reason of this kind of leveling or kick-off.

Based on the previous common grounds identified in the two fields, the idea of cross cutting collaboration starting from the equal leveling of PHAT and TT (as shown in Figure 6 above) is the next step. The starting point is market pull. Market pull/push demonstrates the inter-dependability of new technologies of the PHAT pipeline (in particular HNA and HTA) with TT activity. TT should work two-fold, using its traditional 'opportunity identifying' needs methodology fuelled by market demand and bi-lateral communication through evidence-based advocating for relevant (or upcoming) technologies in its activity with the PHAT organizations and DMs. This can be made possible through public-private partnerships (PPP). According to Laane *et al. *[45], academics, industry (in our case the academic-industrial complex) [23] and government join forces in PPPs to translate basic science into marketable applications with social and economic value. These PPPs can take different forms as it can be on a one-on-one basis or it can involve multiple parties (large to small medium enterprises) or universities to (semi) public or private research organizations and/or involve charities and government. Laane *et al. *[45] further states that PPPs can have physical locations or be virtual (with researchers from different organizations/locations) or it can be entirely different. PPPs are considered to be collaborative (partners work together contributing knowledge/resources), precompetitive (exclusivity not an issue in collaboration) and they are partnerships in which the risks/rewards, funding and intellectual properties are shared. PPPs are supposed to:

i. Bridge the gap between the academic-industrial complex and policy makers

ii. Pool knowledge and resources

iii. Catalyze innovation through translational research

iv. Transmit momentum and gear social/market pull to technology push

v. Multiple investments

vi. Align academic-industrial research agendas with social priorities

vii. Concentrate focus and mass on areas of social priority in which the parties are strong

viii. Increase social and economic returns of basic research

In our case PPPs also involve consultation.

The industry should get involved in the subsequent steps of PHAT, including identifying the current and potential future needs, assessment, impact and prioritizing among others and should involve a feedback loop with communication two-way. This is a good strategy to ensure flexibility and adaptability by both parties leading to higher chances of success both commercially and policy-wise which is principle of communicating vessels. For example, the health/market need and technology could be identified by the industry and possibly via consultation with the end users and DMs (say HNA). After initial research and development, the industry can approach the HTA professionals and DMs with their proposal to fulfill the need and collaborate through the HTA steps and vice versa. This also includes the assessment of the impact the technology will have on society (HIA) in mutual collaboration. In hindsight, this can help covering areas critically ignored by both, the industry and the health care system.

This combination brings into the picture the PHGW, which demonstrates the integration process of Genomics into Public Health and healthcare in general [42]. Beskow *et al. *[42] divides Public Health tasks within three key domains of Public Health (Genomics), viz., Assessment, Policy Development and Assurance. The industry and DMs should systematically and in parallel go through these domains at an early stage within our model and integrate it in their respective strategies of product development and development of evidence-based guidelines. This is in addition to the TT and PHAT inter-dependability, to ensure areas to be covered or gaps are not neglected in their technology development and policy respectively. Thus all critical economic, policy and societal aspects are dealt with pre-emptively which are generally put aside. As a result, the timely as well as effective and efficient transition of relevant technological integration in policy and the health care system can be ensured.

As a consequence of these interactions and setups being developed, it is important that a sustainable infrastructure is in place which can accommodate these interactions since the major component of our strategy involves complex communication. Furthermore, the stakeholders involved should use the concept of this infrastructure to push forward their respective agendas within our 'LAL Model'. Keeping in mind this infrastructure, our model proposes to use/develop and gives rise to the (innovation) network which is an evolving mutual dependency system based on resource relationships in which their systemic character is the outcome of interactions, processes, procedures and institutionalization. Activities within such a network involve the creation, combination, exchange, transformation, absorption and exploitation of resources within a wide range of formal and informal relationships [46,47]. This network is dependent on the VOI (Value of Information). VOI is the maximum price one should pay for knowing the actual value of an uncertainty before deciding on a course of action [43] (see legend of Figure 6). In our case this can be a technology or technique in question to be integrated in health policy and healthcare. According to Oestreich [43] and adapted to our model, the VOI is dependent on three factors, namely relevance to the consumer, processing ability of the current infrastructure (the innovation network-developed through PPP between the academic-industrial complex and government, to understand say its clinical utility or utility in healthcare) and exclusivity to the provider (patent for example). This is a learning process with continued iterations and feedback loops, and requires the ability to learn, assimilate and use knowledge developed elsewhere through a process that involves substantial investments especially of an intangible nature and is called the absorption capacity [47,48].

## Discussion

**Figure 7:**
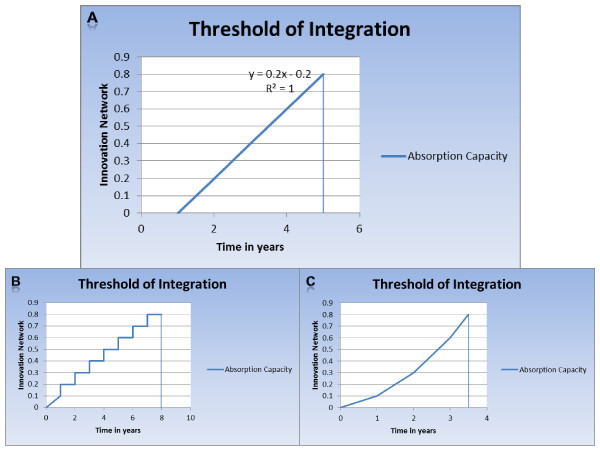
**Absorption Capacity as a function of the innovation network and time**. A hypothetical schematic representation from top counter-clockwise of achieving threshold capacity for technological integration in (A) ideal circumstances, (B) for a newly introduced technology in a non-existing innovation network with the network development hindered through bureaucracy and skepticism of the new infrastructure, (C) of an already tested innovation network moving through a 2^nd ^round of new technological introduction.

Based on our model, we believe the higher the absorption capacity, the higher the possibility of success as can be seen from Figure 7A below. The y-axis represents the progress and development of the innovation network. The numbers on this axis are percentage units of the expansion of the innovation network with 0.1 being 10% and 0.8 being 80%. The x-axis represents time in years which corresponds to the timeline of the TT activity. This graph in ideal situations corresponds with the development of upcoming or new technologies. With regard to relevant technologies already in the market, the same can apply, however the years may be shorter. The gradient represents the absorption capacity, which in our case is the function of the innovation network with respect to time which in turn in dependent on the VOI. The threshold or optimal possibility of the absorption capacity is marked at 80% in (say) 5 years. It can also be noticed that the absorption capacity gradient does not start at year 0 but rather a bit later. As earlier mentioned, the initial stages of TT start in the lab with research and development and the research is motivated on market dynamics and cannot clearly identify with the PHAT's Health Needs Assessment, which in turn is the necessary partner (HTA) of TT, required for the expansion of the innovation network. Therefore progression cannot start at this stage. After this step, within the TT pipeline, the innovation network becomes activated. Below is the proposed idea of the progression:

1. Identification of the health need (market pull/push) by industry and subsequent research and development. We mark this as 0% innovation network.

2. After initial research and development, contact is made with the relevant authorities in HNA/HTA and policy. We consider this 10% development of the innovation network, which starts from 0%.

3. A proposal is developed either jointly (PHAT and industry) or individually (say academia-industry) and it is advocated. Networking is the key here. We consider this 20% development of the innovation network.

4. Initiation of PPP of the industry with policy through the PHAT authorities. Partnership can be in terms of collaborations, knowledge sharing, joint investigations, etc. This is a major milestone; therefore we consider this 40% capacity of the innovation network.

5. Over time the innovation network is developed and expanded as the industry collaborates with decision-makers through the process of the PHAT and TT activity pipelines which can look like a pseudo-merger. We consider this 60% network capacity.

6. Finally, the innovation network has matured enough to integrate relevant technologies into healthcare systems and policy in real time as a consequence and evaluate the impact using HIA. We believe the innovation network at 80% functionality and are considered the threshold of the absorption capacity of this apparatus.

The steps take into consideration the PHGW. The value of 1.0 or 100% seems not feasible as this is a learning process and there will be always room for improvement apart from the fact that there will be always unforeseen internal and external developments. This example shows emerging technology integration via the development from a non-existent innovation network being expanded. The remaining 20% is an ongoing process with feedback loops, within the already now developed innovation network (above 6 steps) for more new or relevant technology integration; thus having a dynamic absorption capacity. This would be considered ideal in the development of the innovation network; however, depending on the technology to be integrated [49] the graph can and will be represented differently for different people/organizations over time as shown in Figure 7B and 7C below.

The involvement of various stakeholders is an essential in order for this model to work. Ideally, government driven regulatory requirements would greatly benefit the model by stimulating the adoption of the model for the right reasons. PPPs, for example, act as a cog in the larger innovation system conveying momentum from one part to the other and are best if the push is from technology and the pull is from social and market demand [45]. As of now the right level of intervention settings and involvement is not ideal. Currently, it is up to the industry to voluntarily advocate and/or implement the model in their settings to benefit. In the process, and as the model indicates, PPPs will bolster the involvement and perhaps future government driven regulatory requirements. Likewise, if decision makers identify the benefits of this model they can voluntarily indicate it to the industry to follow suit. Either way, in realistic setting, one core stakeholder has to take the initiative to kick-start the framework. The core stakeholders to initiate the model are basically the industry (including scientists in other settings) and government decision-makers including PHAT professionals. Other important stakeholders to sustain the process are the patient groups, hospitals and health professionals (including doctors).

It should be emphasized here that since this process involves advocating and PPP, the model's ultimate goal is to benefit the end-consumer, the patient, in a timely and effective manner, and in the process stopping the rightful profit backlog of relevant or upcoming technologies that generally do not reach the patient. As earlier stated, this is dependent on the relevance to the end-consumer combined with the processing ability of the information generated through PPP with exclusivity to the provider. For industry, profit is primary motivation behind innovation research in medicine or healthcare in general. This is not wrong as long as the health of the patient is not compromised through misguided facts of (ir)relevant technologies diffused into the health care apparatus thus affecting the policy-making as a whole. This may or may not arise from market push. In order to avoid this, the industry is encouraged to consider ethical behavior when embarking in this model. To keep this in check, HTA professionals and DMs should always have a third advisor. The first being industry, second being the HTA experts and their sources (neutral or evidence-based recommendations), and the third being a combination of neutral external experts, healthcare providers (e.g. doctors) and patient interest groups. The reason to keep it in this order of recommendations is to encourage the pseudo-merger of TT and PHAT. The industry can advocate for relevant or upcoming technologies in its pipeline based on sound scientific data and health needs through consultation with doctors and patients, in the PHAT pipeline. As a result, the industry needs to positively influence the DMs and HTA professionals to push forward the correct agenda for timely interventions. This can be possible if the industry communicates with the PHAT professionals evidently, hence the first opinion. However, the PHAT infrastructure should not compromise its own recommendation. The third opinion(s) being the most important and the motivation of the target group is self-explanatory as it is the patient group and they aim for improving quality of life through improving health and reducing burden of disease.

As a result, the model promotes an ethical balance between profit and improving health through timely and effective interventions. It should be noted here, however, the classical PHAT apparatus or methodology needs to be compatible with the current trends which is not the case at the moment. Although revised versions of HTA like 'core HTA' [50] have been established, nonetheless, they have not proven sufficient enough to solve current issues. Therefore, a more innovative approach [51] compatible with our model is required, which will be our next step of investigation. Also TT may need to adapt to HTA as well. However, this is within the concept of innovation networks.

A possible limitation of the model may or may not include its lack of addressing the phasing out of an obsolete technology. Depending upon the stakeholder this may be of importance. Phasing out a no longer required technology may not be of concern for an industrial player looking to capitalize on the market with their new technology and seems to be the general perception based on the authors' experience. A very simple example can be microarrays. To our knowledge we have never seen a microarray company phasing out its previous best seller over a newer version (although they phase out the production internally depending upon demand). Rather they simultaneously sell both until the older version fades away. Reason being at the end of the day it boils down the amount of sales. This phasing out of obsolete technologies rather falls on the ears on hospital management, policy makers among others. For them phasing out is equally important as phasing in a new technology. On the other hand it can be argued that industry should pay to a certain extent attention on phasing out obsolete technology parallel to their diffusion of own technologies. This can probably ensure a smooth integration process in healthcare as it will develop a good case for integration of emerging technologies in the healthcare and policy. Our LAL model itself does not address this important issue rather focuses on the phasing in or diffusion of a new technology in healthcare and policy. Nonetheless, our hypotheses is that the same model framework could be used simultaneously while phasing in a new technology, to phase-out an obsolete version, but still has to be experimented, which is like-wise for the phasing in concept itself.

## Conclusions

The LAL model proposes to facilitate and/or speed the valorization process of new and relevant technologies within healthcare systems with less chances of failure through early on involvement of stakeholders (within TT and PHAT). The aforementioned early on involvement of stakeholders can help the industry to come to an advance decision whether or not to continue with the developing/emerging technology or consider upgrading it before introducing it in the market. This can be based on for example the technology's clinical utility (is a measure of the health care value provided by the technology) [52] among others (HTA, etc.) thus saving on resources. Our model has potential to guide valorization in context with integration in health policy and healthcare systems resulting in the timely as well as effective and efficient introduction of relevant genome-based technologies.

Valorization generally neglects the policy side, hence the generally fewer successes of investments by Venture Capitalists in business models targeting healthcare. This new model proposes to bring in fresh components to the valorization process of scientific breakthroughs not only to the bedside but also to the healthcare system as a whole; thus making the process more efficient in real-time, by solving the backlog of relevant technologies in healthcare before they become out-dated due to introduction of upgraded or newer technologies. The added value of this model is that it brings together, as per our knowledge for the first time, two separate entities (TT and PHAT) to early on involvement. As a result this can benefit both health care policy and industrial profit. It encourages a large amount of networking and communication with relevant stakeholders in order to be successful, demonstrates the inter-dependability and parallel initiation of policy and technological innovation. This network builds on the ongoing absorption capacity of the apparatus through an ethical balance between profit and timely interventions for the benefit of the population health. In simplistic terms it is a process of 'Learning, Adapting and Leveling', which in essence is the LAL model.

## List of Abbreviations

BDC: Basic Design Cycle; DM: Decision Makers; FTM: Fish Trap Model; HIA: Health Impact Assessment; HNA: Health Needs Assessment; HTA: Health Technology Assessment; LAL: Learning Adapting Leveling; PHAT: Public Health Assessment Tools; PHGW: Public Health Genomics Wheel; PPP: Public Private Partnership; SWOT: Strengths Weaknesses Opportunities Threats; TT: Technology Transfer; VOI: Value of Information.

## Competing interests

The authors declare that they have no competing interests.

## Authors' contributions

JAL conceived and developed the LAL model with critical input and review from TSIDB, SAM and AB. All authors read and approved the final manuscript.
